# Anti-inflammatory effects in muscle injury by transdermal application of gel with *Lychnophora pinaster* aerial parts using phonophoresis in rats

**DOI:** 10.1186/1472-6882-13-270

**Published:** 2013-10-20

**Authors:** Viviane GC Abreu, Geone M Correa, Thiago M Silva, Humberto S Fontoura, Denise C Cara, Dorila Piló-Veloso, Antônio FC Alcântara

**Affiliations:** 1 Departamento de Química, Instituto de Ciências Exatas, Universidade Federal de Minas Gerais, 31270-901, Belo Horizonte, MG, Brazil; 2 Instituto de Ciências Exatas e Tecnologia, Universidade Federal do Amazonas, 69100-000, Itacoatiara, AM, Brazil; 3 Departamento de Fisioterapia, Universidade Estadual de Goiás, 74705-010, Goiânia, GO, Brazil; 4 Departamento de Morfologia, Instituto de Ciências Biológicas, Universidade Federal de Minas Gerais, 31270-901, Belo Horizonte, MG, Brazil

**Keywords:** *Lychnophora pinaster*, Triterpenes, Flavonoids, Anti-Inflammatory activity, Phonophoresis, Transdermal application

## Abstract

**Background:**

*Lycnophora pinaster* is used by the traditional Brazilian medicine for the treatment of inflammations. Anti-inflammatory activity of *Lycnophora pinaster* was investigated for extracts, fractions, and isolated compounds of their aerial parts. The hexane extract (HE) provided *α*-amyrin, lupeol, mixture of *α*-amyrin and lupeol, mixture of 3-*O*-acetyl-lupeol and 3-*O*-acetyl-pseudotaraxasterol, and mixture of the steroids stigmasterol and sitosterol. The aqueous extract (WE) provided a fraction containing alkaloids (AF) and another one containing phenolic compounds (PF).

**Methods:**

The crude hexane extract obtained from aerial parts of *L. pinaster* was submitted to chromatographic fractionation. The fractionation of PF was performed by preparative HPLC analysis, providing the flavonoid quercetin. The extracts, fractions, and compounds isolated from *L. pinaster* were tested to evaluate the anti-inflammatory activity by experimental model of impact injury, followed by transdermal application of gels with these samples. The application of the gels was performed using phonophoresis in rat paws after induction of muscle injury. Histological analysis was based on scores assigned by the capacity of decreasing the lesion.

**Results:**

HE and WE exhibited anti-inflammatory activity. Some fractions, triterpenes, and steroids also reduced the inflammatory infiltrates caused by muscle injury. Lupeol promoted a significant reduction of inflammation. Quercetin also provided significant results, promoting the greatest decreases in muscle injury.

**Conclusion:**

The results of this work suggest that topical application of triterpenes, steroids and flavonoid significantly decreases the inflammatory process generated by muscle injury. The transdermal application using phonophoresis in rat paws of gel with lupeol and quercetin attenuates the inflammation.

## Background

Species of the genus *Lychnophora* (Asteraceae) are largely used by the traditional Brazilian medicine for the treatment of various diseases, mainly Chagas and inflammatory diseases [1]. *Lychnophora pinaster* Mart, popularly known as “arnica-mineira”, is a native species and widely distributed in the State of Minas Gerais (Brazil). Species of *Lychnophora* exhibit antitumor, antimicrobial [2], anti-pyretic, analgesic [3], anticonvulsant [4], and anti-inflammatory activities and antioxidant property [5]. Recently, we investigated the bactericidal and trypanocidal activities of triterpenes isolated from the leaves, stems, and flowers of *Lychnophora pinaster*[6]. The nonpolar fractions and some isolated triterpenes of the plant exhibited low trypanocidal activity. However, the same fractions and triterpenes exhibited antibacterial action against *Staphylococcus aureus*. Triterpenes also exhibit a large spectrum of antitumor properties which are likely related to their anti-inflammatory activity [7].

The present work describes the evaluation of the anti-inflammatory activity of hexane and aqueous extracts (HE and WE, respectively) obtained from the aerial parts of *L. pinaster.* Triterpenes and steroids isolated from HE and alkaloid (AF) and phenolic fractions (PF) obtained from WE were also evaluated. The chemical identification of the isolated compounds was carried out by spectroscopic methods mainly based on ^1^H and ^13^C NMR data.

A large number of studies in the literature describes experimental models for evaluation of systemic anti-inflammatory activity. A problem of systemic models involves the first-pass metabolism in the liver. This metabolism is common in the case of orally administered drugs, promoting their degradation and reduction in bioavailability [8,9]. Consequently, anti-inflammatory drugs may be few effective and cause serious side effects when they are orally administered [10,11]. On the other hand, the use of topical formulations offers several advantages and exhibits efficient therapeutic results in relation to other modalities of administration [12,13]. Therefore, anti-inflammatory assays for the plant material were performed in the present work by the method of transdermal application using phonophoresis in rats.

## Methods

### General procedures

Hexane, methanol, trifluoracetic acid, ammonium hydroxide, ethyl acetate, ethyl ether, and hydrochloric acid were purchased from Merck or Sigma-Aldrich. Dexamethasone was purchased from Aché. Ketamine and xylazine were purchased from Birch World. All solvents/reagents were analytical grade and used without further purification.

The ^1^H and ^13^C NMR spectra at 400.129 and 100.613 MHz, respectively, were performed on a Bruker DRX400 AVANCE spectrometer, using CDCl_3_ or MeOD as solvent, with direct or inverse probe and a field gradient. The chemical shifts were registered in ppm (*δ*) relative to TMS as the internal standard. The coupling constants (*J*) were registered in Hertz.

HPLC analyses in analytical scale were carried out in a Shimadzu liquid chromatograph, consisting of two pumps (LC-20AT), detector UV/VIS (SPD-20A), software LCsolution v. 1.21 (Shimadzu), and column ODS Hypersil (C_18_), 250 mm × 4.6 mm × 5 mm (Supelco). Aliquots of each sample were analyzed by HPLC, in isocratic mode, using as mobile phase a mixture of 20% methanol and 80% solution of water:trifluoracetic acid (99.5:0.5 v/v; pH 4.3) during 30.0 min. The injection volume (20 *μ*L) was manually injected at a flow rate of 1.0 mL/min.

Preparative HPLC analyses were performed in a Shimadzu liquid chromatograph, consisting of a pump (LC-10AV), detector UV/VIS (SPD-10AV), software PC/Chrom^+^ (u8A Scientific) column Dyna-Max Microsorb (C_18_) 10C-5250 × 10.0 mm (Varian), and a guard column. The isocratic mobile phase was the same as described above for HPLC analyses in analytical scale, with injection volume of 2 mL, manually applied and flow rate of 4.7 mL/min. The chromatographic data were obtained, analyzed, and stored at 280 nm. After preparative procedure, the samples were lyophilized using the equipment TermoFisher FR-Drying Digital Unit (Scientific). The samples were stored at -18°C until required for experiments.

### Phytochemical methodology

#### Plant material

The plant material of *Lychnophora pinaster* was collected in April 2009 at Moeda Mountain, in the City of Nova Lima, State of Minas Gerais (Brazil). A voucher specimen of *L. pinaster* was deposited in the Herbarium of the Instituto de Ciências Biológicas of the Universidade Federal de Minas Gerais, under the code BHCB: 24,322. The plant was identified by Dr. J. Semir (Departamento de Botânica, Universidade Estadual de Campinas). The aerial parts (leaves, flowers, and stems) of the plant were dried at room temperature until constant weight was achieved (about one week) and finally powdered.

#### Fractionation of the hexane extract

The powdered plant material (5609.20 g) was submitted to extraction with hexane at room temperature, obtaining the crude hexane extract (HE; 92.97 g). The chromatographic fractionation of HE provided the triterpenes *α*-amyrin, lupeol, mixture of triterpenes 3-*O*-acetyl*-*lupeol and 3-*O*-acetyl*-*pseudotaraxasterol, and mixture of steroids stigmasterol and sitosterol, as previously described in the literature [6].

#### Fractionation of aqueous extract (WE)

The powdered plant material (200.00 g) was submitted to decoction with water for 2 h at 60°C, obtaining the aqueous extract (WE; 2.9 g). Ammonium hydroxide was added to WE until pH 10.0-11.0 and then 3:1 ethyl acetate:ethyl ether solution (100 mL) was also added. The organic and aqueous phases were separated. Hydrochloric acid was added to the aqueous phase until pH 1.0-2.0 and 3:1 ethyl acetate:ethyl ether solution (100 mL) were again added. The organic and aqueous phases were separated. After organic solvent evaporation, the phenolic fraction was obtained (PF; 264.0 mg). Chemical identification test indicated presence of phenolic compounds in this fraction. On the other hand, the aqueous phase provided the alkaloid fraction (AF; 210.0 mg). Chemical identification test indicated presence of alkaloid in this fraction [14].

PF was submitted to fractionation by preparative HPLC analysis (1:4 methanol–water solution; flow 4.7 mL/min), providing quercetin (**1**) [15]: ^1^H NMR (400 MHz; CDCl_3_) *δ*_H_ 7.74 (d, *J* = 2.1 Hz; H-2′), 7.65 (dd, *J* = 8.5 and 2.1 Hz; H-6′), 6.92 (d, *J* = 8.5 Hz; H-5′), 6.41 (d, *J* = 2.1 Hz; H-8), and 6.20 (d, *J* = 2.1 Hz; H-6); ^13^C NMR (100 MHz; CDCl_3_) *δ*_C_ 177.5 (C-4), 165.7 (C-2), 162.6 (C-9), 158.3 (C-7), 148.9 (C-3′), 148.1 (C-5), 146.4 (C-4′), 137.4 (C-3), 124.3 (C-1′), 121.8 (C-2′), 116.1 (C-5′), 115.8 (C-6′), 104.7 (C-10), 99.5 (C-8), and 94.6 (C-6).

### Anti-inflammatory tests

#### Animals

Experiments were performed on male rats (200.0 to 300.0 g) purchased from Bioagri Laboratórios LTDA (City of Planaltina-DF, Brazil). The animals were kept in plastic cages at 22 ± 2°C on a 12 h light/dark cycle with free access to pellet food and water, according to international guiding principles for biomedical research involving animals. The animals were acclimatized for four days before beginning the experiments.

#### Obtaining muscle injury

Ketamine and xylazine (80.0 and 10.0 mg/kg body weight, respectively) were diluted in 1 mL of saline solution and employed to intraperitoneal (i.p.) anesthesia. Animals were separated into groups: a negative control group (without treatment), a positive control group (with standard treatment, animals treated with dexamethasone 0.1%), and 10 groups with treatment (animals treated with the crude extracts, the fractions, and the pure compounds). In each group of animals the treatment was performed once a day for three consecutive days (24, 48, and 72 h).

The animals suffered muscle injury which was generated by the impact of a loose weight of 300.0 g at 30.0 cm high in the hamstring and calf backs. The trichotomy on bilateral gluteal region of all injured paws was performed by application of gels previously prepared. After 24 h of the treatment, the animals were anesthetized and sacrificed for muscle collection. The other sacrifices were repeated after 48 and 72 h. The entire experiment was approved by the Ethics Committee on Animal Use of the Universidade Federal de Minas Gerais (CETEA/UFMG), under protocol number 222/11.

#### Treatment

Twelve groups of rat (n = 3 for each group) were subjected to muscle injury in both the paws (right and left). After injury, the right paw of each animal was treated with therapeutic ultrasound in pulsed mode at a frequency of 1 MHz, with intensity 0.5 W/cm^2^ for 9 min [16]. The treatment was performed using as couplant in the therapeutic ultrasound crude extracts (3.0% m/m), the fractions (0.5% m/m), the pure compounds (0.5% m/m), and the positive control, dexamethasone, 0.1% m/m [17], which were incorporated in the form of carbopol gel. The left paw of each animal was used as negative control and received the internal coupling head at the same time. However, the phonophoresis equipment was off and the gel was not applied, using the same protocol employed to the treated paw. The results were expressed by the ability to decrease the inflammatory infiltrate.

#### Histological analysis

After 24, 48, and 72 h of the treatment, the rats were anaesthetized and sacrificed. The paw tissues were removed, fixed in 10% formalin in PBS, embedded in paraffin, and cut into 4 *μ*m thickness sections. The sections were stained by hematoxylin-eosin for histological analysis. A representative area was selected for qualitative light microscopic analysis of the inflammatory cellular response with a 10× objective [18].

## Results and discussion

**Figure 1:**
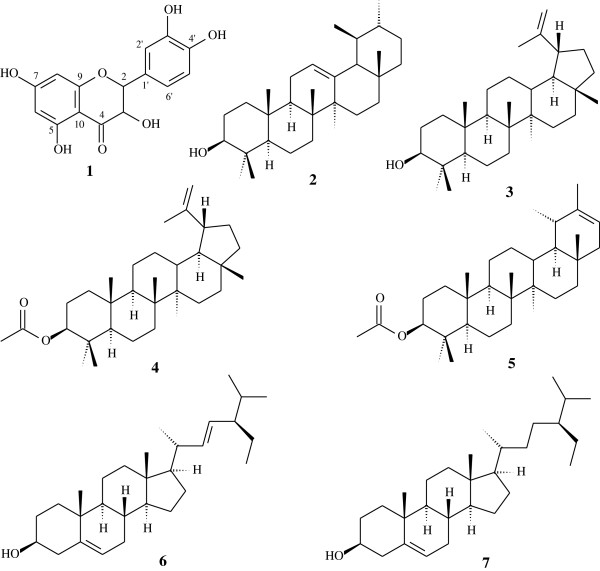
**Chemical structure of the compounds isolated from the aerial parts of *Lychnophora pinaster *** **Mart.**

The fractionation of the PF resulted in the isolation of quercetin (**1**). The phytochemical study of the hexane extract obtained from the aerial parts of *L. pinaster* resulted in the isolation of *α*-amyrin (**2**), lupeol (**3**), mixture of 3-*O*-acetyl*-*lupeol (**4**) and 3-*O*-acetyl*-*pseudotaraxasterol (**5**), and mixture of stigmasterol (**6**) and sitosterol (**7**), see Figure 1[6].

**Figure 2:**
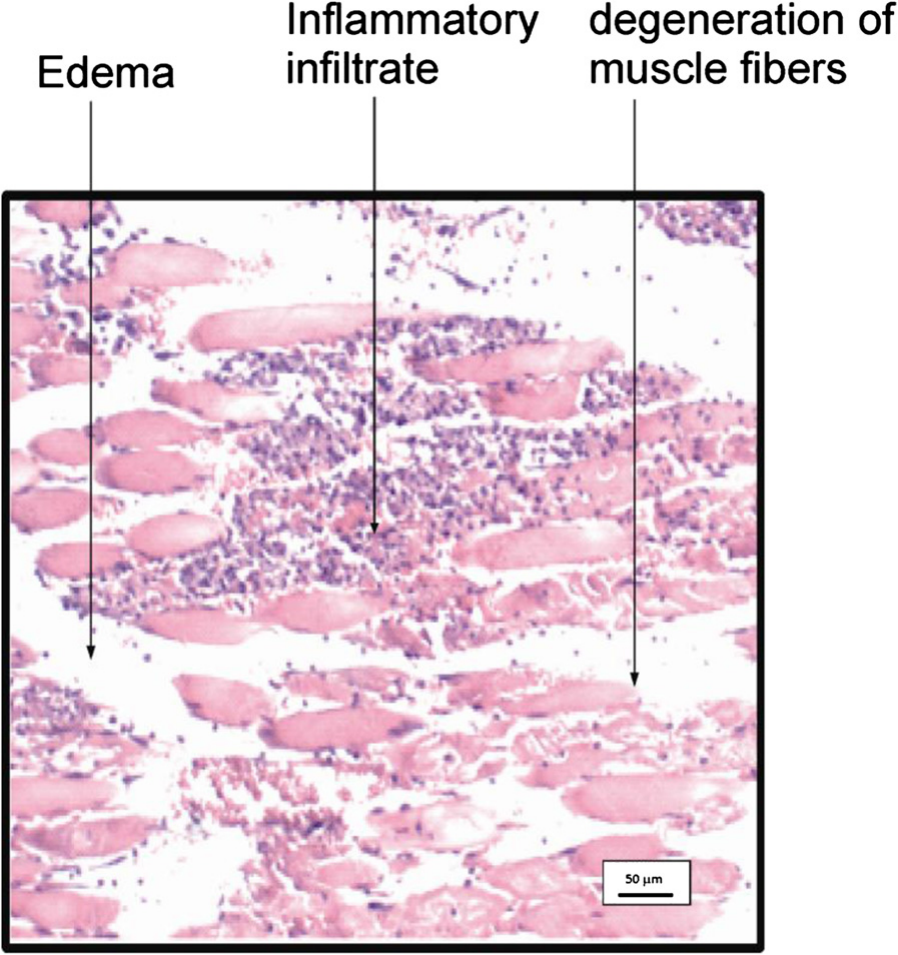
**Histological aspect of the injured muscle of rat paws without treatment, after 24 h.** Regions of inflammatory infiltrate, edema, and degeneration of muscle fibers are indicated by arrows.

The anti-inflammatory activity of extracts, fractions, and triterpenes of the plant material were based on histological analysis to characterize inflammatory infiltrates. Figure 2 shows the histological aspect of the injured muscle of rat paw without treatment (negative control group) after 24 h of the muscle injury. The section exhibits a large regions of edema (in white) and inflammatory infiltrate (purple dots spread on the section). Muscle tissues are represented by pink spots. A normal pattern of muscle tissue should show pink continuous fibers. However, the fragmentation of muscle tissue observed in Figure 1 indicates significant degeneration of muscle fibers [19].

**Figure 3:**
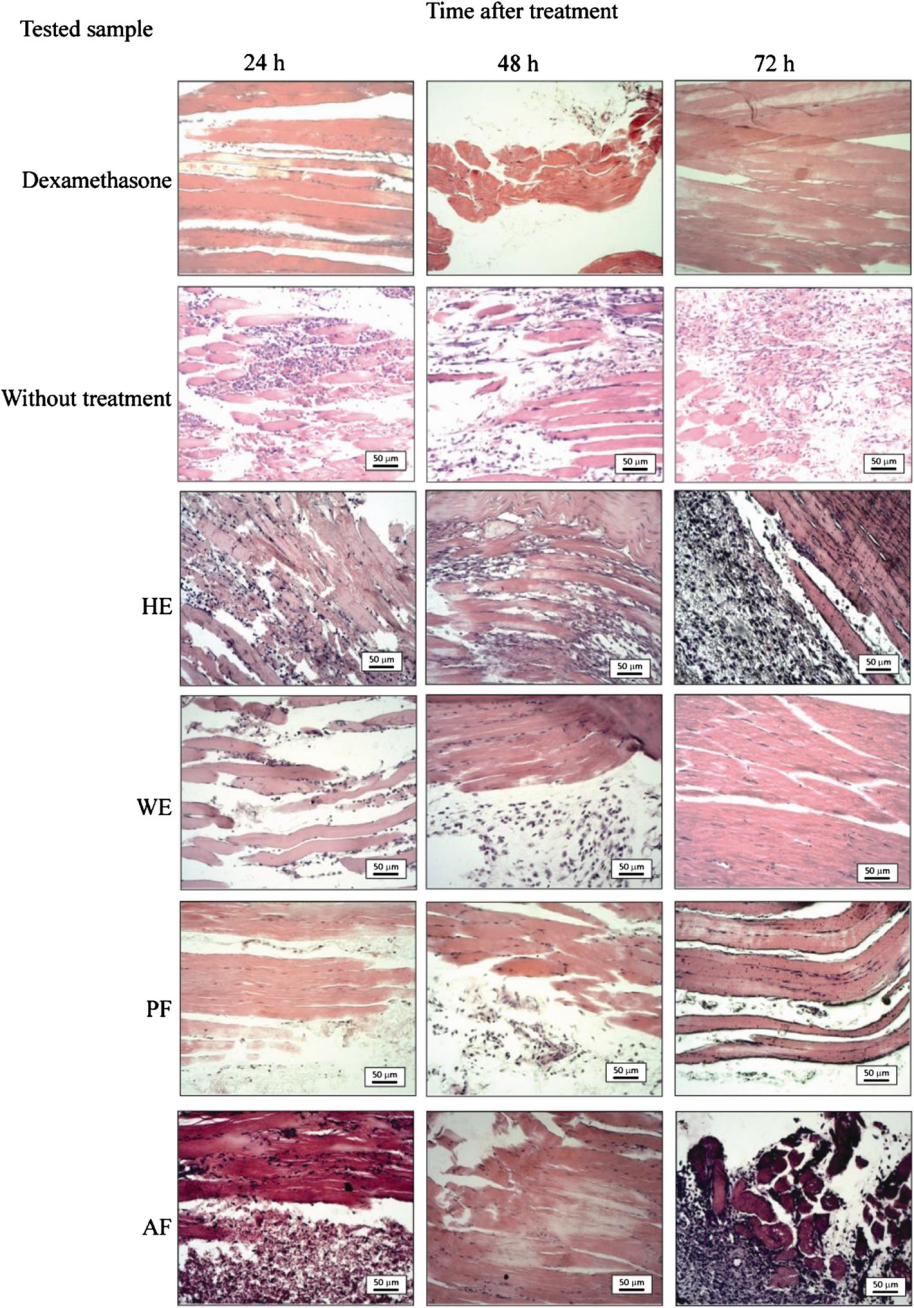
**Histological aspect of the injured muscles of rat paws without treatment and treated, after 24, 48, and 72 h of the application of gels with dexamethasone, hexane extract (HE), aqueous extract (WE), phenolic fraction (PF), and alkaloid fraction (AF) of *L. pinaster*.**

Figure 3 shows histological aspect of the injured muscles of the rat paws without treatment and those treated with dexamethasone, extracts, and fractions of *L. pinaster*. The sections of the rat paws without treatment exhibit a few significant decrease of the inflammatory infiltrate after the injury, presenting moderate injury at 24 h, mild to moderate injury at 48 h, and mild injury at 72 h. In this time period, edema and degeneration of muscle fibers are increased of moderate to severe injury. Dexamethasone is a potent anti-inflammatory drug [17,20]. The injured muscles of the rat paws treated with dexamethasone exhibit mild inflammatory infiltrate at 24 and 48 h, decreasing to without injury at 72 h. Edema is moderate to mild and degeneration of muscle fibers is mild at 24 h. Both the symptoms are severe and mild at 48 and 72 h, respectively (see Table 1).

**Table 1:** Assigning scores of anti-inflammatory activity-based control group*

**Sample treatment**	**Time after**		
	**24 h**	**48 h**	**72 h**
Control without treatment	3	2	1
Control with standard treatment (Dexamethasone)	1	1	0
Hexane extract (HE)	2	1	3
Aqueous extract (WE)	1	3	0
Phenolic fraction (PF)	1	2	1
Alkaloid fraction (AF)	3	1	3
Quercetin	1	0	0
*α*-Amyrin	2	1	2
Lupeol	1	0	0
Lupeol and *α*-amyrin mixture	2	1	0
3-*O*-Acetyl-lupeol and 3-*O*-acetyl-pseudotaraxasterol mixture	2	3	2
Stigmasterol and sitosterol mixture	2	1	0

*The comparison between the groups follows injure score: 0–without injury; 1–mild; 2–mild to moderate; 3–moderate; 4–moderate to severe; 5–severe.

The assay with HE exhibits mild to moderate inflammatory infiltrate at 24 h, decreasing at 48 h, increasing to moderate at 72 h (Figure 3). These results suggest a complexity of different synergic and antagonistic effects of constituents of HE in their anti-inflammatory action. On the other hand, edema and degeneration of muscle fibers are remained mild during the investigated period.

The histological aspect of the injured muscles treated with WE (Figure 3) exhibits severe edema at 24 and 48 h, reducing this symptom to mild at 72 h. Degeneration of muscle fibers is mild during this time period. Inflammatory infiltrate is mild at 24 h and moderate at 48 h. However, a significant decrease of the inflammatory infiltrate can be observed at 72 h. These results justified the fractionation of the plant extract to investigate the anti-inflammatory activity of phenolic and alkaloid fractions. AF exhibits a moderate injury at 24 h and mild injury at 48 h, increasing inflammatory infiltrate at 72 h. Edema and regeneration of muscle fibers are mild at 24 and 48 h, increasing to moderate/severe at 72 h. PF exhibits severe edema and mild degeneration of muscle fibers from 24 to 72 h. The inflammatory infiltrate is mild at 24 h, mild to moderate at 48 h, and finally mild at 72 h, indicating a high proportion of active components in this fraction, justifying its fractionation by HPLC.

**Figure 4:**
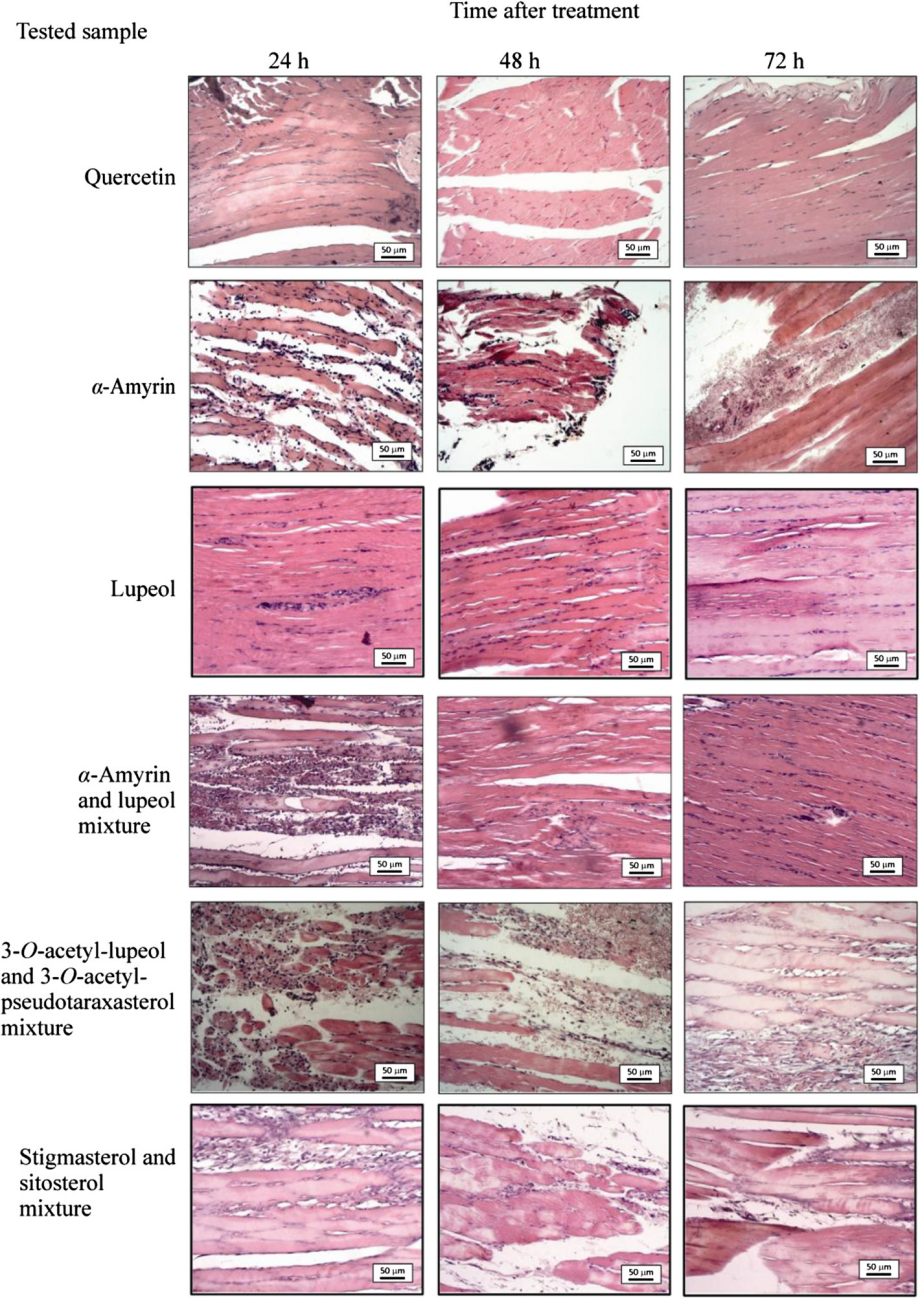
**Histological aspect of the injured muscles of the rat paws treated after 24, 48, and 72 h of the application of gels with isolated compounds of *L. pinaster*.**

Figure 4 shows histological aspect of the injured muscles of the rat paws treated with phytoconstituents isolated from aerial parts of *L. pinaster*. The sections of rat paws treated with quercetin exhibit mild edema without degeneration of muscle fibers from 24 to 72 h. Inflammatory infiltrate is mild at 24 h, not being observed at 48 and 72 h. The anti-inflammatory action of PF, as verified in Figure 3, can be attributed to quercetin.

The treatment with *α*-amyrin has as result severe to moderate edema and degeneration of muscle fibers at 24 and 48 h, but it is only moderate at 72 h. The inflammatory infiltrates are mild to moderate in this time period. The treatment with lupeol has as result a muscle tissue with insignificant occurrences of edema, degeneration of muscle fibers, and inflammatory infiltrate. It can be observed few inflammatory infiltrate at 24 h and few edema at 48 h. The mixture of *α*-amyrin and lupeol provides intermediate results of their inflammatory actions, except at 72 h, when edema, degeneration of muscle fibers, and inflammatory infiltrate are not observed in the sections. A mixture of the triterpenes *α*-amyrin and lupeol (3:1 ratio) promoted a decrease in inflammatory lesions and may be associated with the later inflammatory mediators. The triterpene lupeol promoted a faster and evident improvement in the inflammation, suggesting that *α*-amyrin in mixture with lupeol may exert some antagonistic action in the treatment process, since the concentration of *α*-amyrin in the mixture was larger. The low inflammatory activity suggested for HE (Figure 3) may be related to a low concentration of triterpenes in this extract, mainly the triterpene lupeol, which exhibits significant anti-inflammatory activity.

The rat paws treated with mixture of 3-*O*-acetyl*-*lupeol and 3-*O*-acetyl*-*pseudotaraxasterol exhibit severe to moderate edema and degeneration of muscle fibers. The inflammatory infiltrate is mild to moderate at 24 h, moderate at 48 h, and mild to moderate at 72 h. Similarly, the treatment with mixture of stigmasterol and sitosterol has as result severe to moderate edema and degeneration of muscle fibers from 24 to 72 h. However, inflammatory infiltrates are moderate at 24 h, mild at 48 h, and nonexistent at 72 h. These results may be related to a higher activity of stigmasterol, since the ratio of stigmasterol and *β*-sitosterol in the mixture is 2.5:1.0.

As result, the damaging stimulus generated a pathological state in the rat paw, histologically characterized by edema, hemorrhage, and inflammatory infiltration at 24 h after treatment. The inflammatory infiltrate was predominantly polymorphonuclear and located between muscle fibers. After 48 h, it is observed a predominance of infiltration in perimuscular regions with early organization marked by the presence of extracellular matrix and fibrin. After 72 h, it is still observed infiltration usually marked by mononuclear cells and conjunctive fibers. It is possible to observe an intense inflammation in all paws without receiving no treatment and the presence of some spots generalized edema and hemorrhage.

## Conclusions

The results of this work suggest that topical application of triterpenes, steroids, and flavonoid significantly decreases the inflammatory process generated by muscle injury. The significant results were obtained with the triterpene lupeol and the flavonoid quercetin, having an improvement of inflammation from 24 to 72 h, with a significant reduction in edema and inflammatory infiltrate. The transdermal application using phonophoresis in rat paws of gel with lupeol and quercetin attenuates the inflammatory profile. Therefore, good anti-inflammatory potential can be proposed for topical application of these substances, compared to standard dexamethasone.

## Competing interests

The authors declare that they have no competing interests.

## Authors’ contributions

VGCA, GMC, TMS, and HSF lead the phytochemical and biological activity studies; VGCA and AFCA wrote the manuscript; DCC, DPV, and AFCA supervised the study. All the authors read and approved the final manuscript.

## Pre-publication history

The pre-publication history for this paper can be accessed here:

http://www.biomedcentral.com/1472-6882/13/270/prepub
